# Dissipationless layertronics in axion insulator MnBi_2_Te_4_

**DOI:** 10.1093/nsr/nwad262

**Published:** 2023-10-10

**Authors:** Shuai Li, Ming Gong, Shuguang Cheng, Hua Jiang, X C Xie

**Affiliations:** School of Physical Science and Technology, Soochow University, Suzhou 215006, China; Institute for Advanced Study, Soochow University, Suzhou 215006, China; International Center for Quantum Materials, School of Physics, Peking University, Beijing 100871, China; Department of Physics, Northwest University, Xi’an 710069, China; Institute for Advanced Study, Soochow University, Suzhou 215006, China; Interdisciplinary Center for Theoretical Physics and Information Sciences, Fudan University, Shanghai 200433, China; International Center for Quantum Materials, School of Physics, Peking University, Beijing 100871, China; Interdisciplinary Center for Theoretical Physics and Information Sciences, Fudan University, Shanghai 200433, China; Hefei National Laboratory, Hefei 230088, China

**Keywords:** axion insulator MnBi_2_Te_4_, layertronics, antiferromagnetic domain wall, dissipationless transport

## Abstract

Surface electrons in axion insulators are endowed with a topological layer degree of freedom followed by exotic transport phenomena, e.g., the layer Hall effect. Here, we propose that such a layer degree of freedom can be manipulated in a dissipationless way based on the antiferromagnetic $\rm {MnBi_2Te_4}$ with tailored domain structure. This makes $\rm {MnBi_2Te_4}$ a versatile platform to exploit the ‘layertronics’ to encode, process and store information. Importantly, the layer filter, layer valve and layer reverser devices can be achieved using the layer-locked chiral domain wall modes. The dissipationless nature of the domain wall modes makes the performance of the layertronic devices superior to those in spintronics and valleytronics. Specifically, the layer reverser, a layer version of the Datta–Das transistor, also fills up the blank in designing the valley reverser in valleytronics. Our work sheds light on constructing new generation electronic devices with high performance and low-energy consumption in the framework of layertronics.

## Teaser

Dissipationless layertronics of the axion insulator can be achieved using the antiferromagnetic domain walls of MnBi_2_Te_4_.

## INTRODUCTION

The invention and application of transistors have achieved great success in manipulating the electronic charge degree of freedom [1]. With an increasingly deepened understanding of electronic transport, concepts of designing high-performance devices using the internal degrees of freedom of electrons have sprung up. Among these, spintronics [2–14] and valleytronics [15–26] are the best-known paradigms. Spintronic devices, such as the spin filter [2,3], spin valve [12,13] and spin transistor [4,5], and valleytronic devices, such as the valley filter [15,21,23] and valley valve [15,17], were theoretically raised. Tremendous experimental efforts have been devoted to realizing these promising devices in various materials [26–30]. Nevertheless, from the application level, the energy consumption strongly limits their performance. For many spintronic or valleytronic devices, the bulk carriers are inevitably scattered by the impurities, which dramatically lower their efficiency and increase their energy consumption [17,24,31,32]. It is thus highly desirable to exploit a new degree of freedom of electrons that is robust against disorder.

Recently, a new type of Hall effect, dubbed the layer Hall effect, was reported in the even-layered antiferromagnetic (AFM) axion insulator (AI) $\rm {MnBi_2Te_4}$ [33]. The layer-locked Berry curvature endows the surface electrons in the AI with a topological nontrivial degree of freedom. Hopefully, one can construct devices using such a layer degree of freedom [34,35], and the encoded information can be easily read out through layer-resolved transport measurements [33,36]. However, current experimental and theoretical advances are unable to utilize topologically protected excitations to manipulate the layer degree of freedom in AFM $\rm {MnBi_2Te_4}$ dissipationlessly [33,36–39].

In this work, motivated by recent experimental progresses [33,36,40–58], we highlight that such a layer degree of freedom can be manipulated in a dissipationless way using the domain wall (DW) modes of AFM $\rm {MnBi_2Te_4}$ [59–61]. In parallel to spintronics and valleytronics, we introduce ‘layertronics’, which is committed to designing scalable, low-dissipation devices to encode, process and store information using the layer degrees of freedom of AIs. Accordingly, we propose three of the most important layertronic devices: layer filter, layer valve and layer reverser. Firstly, the layer filter can be constructed through a single DW of AFM $\rm {MnBi_2Te_4}$. We demonstrate that the injected layer-unpolarized current can be successfully filtered to be fully layer polarized and to transport dissipationlessly. The efficiency of the layer filter is robust against disorder and irregularity of the DW structures. Then, the layer valve can be achieved using two pairs of domains. By controlling the chemical potential of different domains, the layer valve can turn on or off the layer-polarized currents. Our results verify that the layer valve has high on:off ratio and is also robust against disorder. Finally, the layer reverser can be constructed utilizing the Chern insulator (CI) phase of the ferromagnetic (FM) $\rm {MnBi_2Te_4}$ [36,50]. The chiral DW mode in an AI-CI-AI heterostructure connects the top and bottom surfaces, and thus can reverse the layer-polarized current dissipationlessly. The layer reverser bears similarity to the Datta–Das transistor in spintronics [62–64], and also fills up the blank of ‘valley reverser’ in valleytronics.

### Model hamiltonian and the layer filter

We model AFM $\rm {MnBi_2Te_4}$ by a three-dimensional (3D) topological insulator (TI) with antiparallel layer magnetization [41,65]. The Hamiltonian is *H* = *H*_0_ + *H*_mag_, where *H*_0_ represents a four-band TI [66,67] with $H_0(k)=\sum ^4_{i=1} d_{i}(\mathbf {k}) \Gamma _i,$ where *d*_1_(**k**) = *A*_1_*k_x_, d*_2_(**k**) = *A*_1_*k_y_, d*_3_(**k**) = *A*_2_*k_z_*, $d_4(\mathbf {k})=M_0-B_1k^2_z-B_2(k^2_x+k^2_y)$ and Γ_*i*_ = *s_i_* ⊗ σ_1_ (*i* = 1, 2, 3) and Γ_4_ = *s*_0_ ⊗ σ_3_ are Dirac matrices. Hereafter, we choose *M*_0_ = 0.3, *A*_1_ = *A*_2_ = 0.55 and *B*_1_ = *B*_2_ = 0.25 [65]. Hamiltonian *H*_mag_ gives the Zeeman splitting *H*_mag_ = *M*(*z*)*s*_3_ ⊗ σ_0_, where *M*(*z*) = ±*M_z_* is the magnetization of each individual layer along the *z* direction, which is fixed as *M_z_* = 0.05, unless specified otherwise (in our calculations, the side surface states are gaped out in order to exclude their transport contribution for a realistic large-size $\rm {MnBi_2Te_4}$ sample). For the AFM AI, the magnetization alignments of two adjacent layers are antiparallel. In the online supplementary material, we performed calculations using realistic material parameters with *M*_0_ = −0.12 eV, *A*_1_ = 2.70 eV Å, *A*_2_ = 3.20 eV Å, *B*_1_ = −11.90 eV Å ^2^, *B*_2_ = −9.40 eV Å ^2^ and *a* = 1.35 nm [41,68,69]. We adopt *M_z_* = 0.04 eV, which induces a surface magnetic gap of about 0.08 eV and is consistent with the theoretical and experimental studies (ranging from 0.05 to 0.10 eV) of $\rm {MnBi_2Te_4}$ [40–43,70–72].

**Figure 1.**
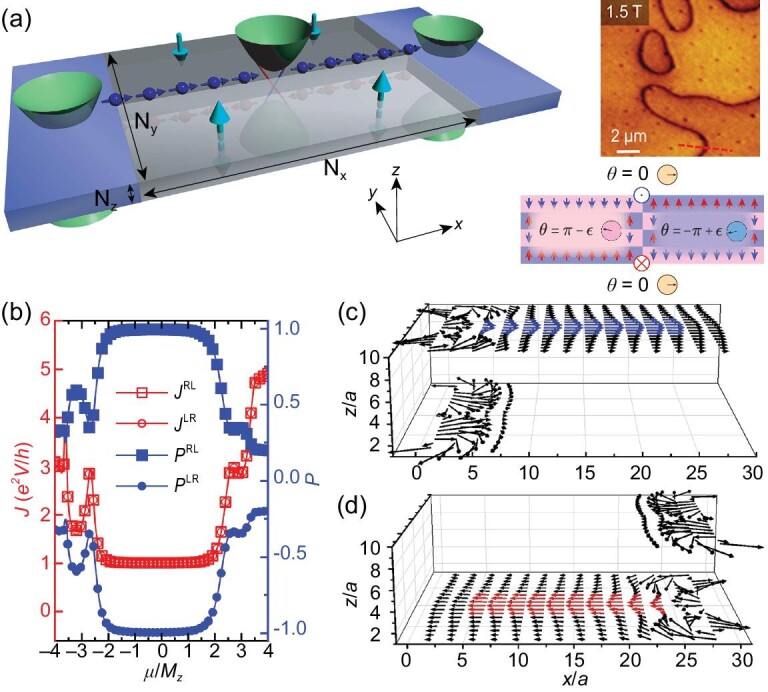
(a) Left panel: schematic of a layer filter. Green hyperbolic bands denote the dispersion of the gapped surface states. Red (blue) line represents the top (bottom) DW modes. Left and right terminals are AFM AIs with the Fermi energy *E_F_* located in the surface states. Right panel: the magnetic force microscopy image of the domain structure in AFM AI $\rm {MnBi_2Te_4}$ (taken from [45]), below which is a sketch of the layer magnetization of AIs and the direction of chiral DW modes. The circled arrows on each domain indicate the axion angle θ. (b) Transmission current *J* and layer polarization *P* of the layer filter versus chemical potential μ (in units of *M_z_*) of the central region. (c and d) show local current distributions for the top and bottom layer filters (μ = 0.02), respectively. Blue (red) arrows emphasize conducting current near the DWs. The model parameters are *N_z_* = 10, *N_y_* = *N_x_* = 20 and the terminal chemical potential is 0.2, with *a* the lattice constant that measures the distance between two adjacent septuple layers.

The layer filter is the most fundamental element of layertronic devices, which generates a fully layer-polarized current. As sketched in Fig. 1(a), the layer filter is constructed through a single DW of AFM $\rm {MnBi_2Te_4}$. The magnetization direction is illustrated by the perpendicular arrows. Because of the AFM magnetization, gaps are opened on Dirac surface states of the AI, leading to half-quantized Hall conductances ±*e*^2^/2*h* on the top or bottom surfaces; see [73,74] and the online supplementary material. The AFM order introduces the topological magneto-electric coupling term $\Delta \mathcal {L}=({\alpha \theta }/{4\pi ^2})\mathbf {E}\cdot \mathbf {B}$, where α is the fine structure constant and θ is the axion angle [75–77]. When the two different types of AFM orders meet to form a heterostructure, the metallic interface states of the AIs are gapped [78,79], leaving the bulk insulated. The crossing lines of the three phases, i.e. the two types of AFM AIs (with θ = ±π) and the vacuum (θ = 0), can be viewed as line defects of the θ field [80]. Its winding direction is illustrated in Fig. 1(a). Consequently, chiral modes appear on the vortices of θ due to the Callan–Harvey anomaly [81]. These chiral DW states are locked with the layer degree of freedom and facilitate the generation of the layer-polarized transmission current.

The AFM DWs can be easily achieved experimentally in $\rm {MnBi_2Te_4}$, as shown by the magnetic force microscopy image of the domain structure in AFM AI $\rm {MnBi_2Te_4}$ [right panel of Fig. 1(a)][45] (in bilayer graphene, similar valley filter devices have been realized in naturally formed DWs [82]). Moreover, a recent experiment has also successfully realized the AFM DWs in eight-septuple-layered $\rm {MnBi_2Te_4}$ through helicity-dependent optical control [83]. Besides, the AFM order in $\rm {MnBi_2Te_4}$ can be reversed through the **E** · **B** term [33], meaning that it is also possible to fabricate the DWs in a gate-controllable manner; see the online supplementary material. The external lead is realized by locating Fermi energy *E_F_* of the AI into the surface bands, but kept inside the bulk gap to ensure that only surface currents are engaged. To quantitatively investigate the layer polarization of the current, we define the polarization coefficient as [15,24]


(1) \begin{eqnarray*}
P(E_F)=\frac{J_t(E_F)-J_b(E_F)}{J_t(E_F)+J_b(E_F)},
\end{eqnarray*}

where the layer-resolved currents are given by


(2) \begin{eqnarray*}
J_{t(b)}(i_x,E_F)=\sum _{\begin{array}{c} \scriptstyle 0\leqslant i_y\leqslant N_y\\
\scriptstyle i_z>(\leqslant )N_z/2\end{array}} J_{x}(i_x,i_y,i_z,E_F).
\end{eqnarray*}

Here, the layer filter considered is a slab with size *N_x_* × *N_y_* × *N_z_* [Fig. 1(a)], *J_x_*(*i_x_, i_y_, i_z_, E_F_*) is the *x* component of the local current on site (*i_x_, i_y_, i_z_*) under bias *V* and *J_t_* (*J_b_*) represents the transmission current near the top (bottom) layer. We numerically calculate the transmission current *J* = *J_t_* + *J_b_* and layer polarization *P* versus chemical potential μ of the central region, where μ = *E_F_* − *E*_0_ measures the difference between *E_F_* and the energy of the Dirac point *E*_0_ [red line with open squares and blue line with filled squares in Fig. 1(b)]. In the gap of the surface states, *J* = *e*^2^*V*/*h* is quantized, meaning that the filtering process is dissipationless and the transmission current becomes fully layer polarized on the drain terminal with *P* = +1. We further calculate the local current distribution to visualize the layer polarization. As shown in Fig. 1(c), the layer filter only permits the top layer current (indicated by the blue arrows), blocking the bottom layer current, so we call it the top layer filter (TLF). Similarly, we can also obtain the bottom layer filter (BLF) by simply reversing the source and drain. The transmission current *J* and polarization *P* [Fig. 1(b)] show that inside the gap, the transmission current is still quantized (*J* = *e*^2^*V*/*h*), but fully layer polarized with *P* = −1. The local current distribution also verifies that the current only goes through the bottom surface, as shown in Fig. 1(d). The layer polarization of the current can be detected through the scanning microwave impedance microscopy images. The measured current signal on the top layer of $\rm {MnBi_2Te_4}$ for the TLF will be much stronger than that for the BLF, although the conductances for the two cases are both quantized [54,55,84].

**Figure 2.**
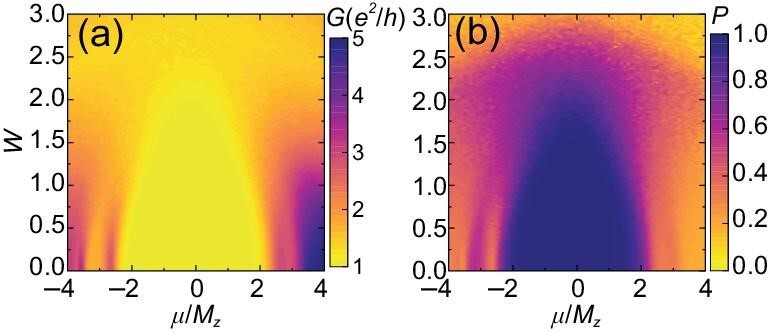
(a and b) *G* and *P* of the TLF as functions of μ and *W*. Other parameters are the same as in Fig. 1.

The performance of electronic devices is usually suppressed by disorders [24,32]. Here, we show that the efficiency of the layer filter is robust against weak disorders. We introduce a random on-site potential term *H*_dis_ = *U*(*r*)*s*_0_ ⊗ σ_0_, where *U*(*r*) is a local potential uniformly distributed within [−*W*/2, *W*/2]. The differential conductance *G* = *J*/*V* and layer polarization *P* of the TLF versus μ and disorder strength *W* are plotted in Fig. 2. For small *W, G* remains quantized and the current remains fully polarized (*P* = 1) for a μ window. By increasing *W*, the μ window with quantized *G* for the TLF shrinks, but still survives under strong disorder strength (such as *W* = 1; see Fig. 2) due to the robustness of the topologically protected chiral DW modes.

Though the key physics are captured by the model study, more realistic considerations can facilitate the experimental realization of the layer filter; see the online supplementary material. Calculations using realistic material parameters of $\rm {MnBi_2Te_4}$ show that an ideal DW (DW width *W_D_* = 0) can act as a filter when *E_F_* is inside the surface gap (≈2*M_z_* = 0.08 eV). The finite width of the DW can induce trivial one-dimensional sub-bands, of which the gap is proportional to 1/*W_D_* and may lie within the surface gap. Nevertheless, as we estimated using realistic material parameters, a sub-band gap larger than 10 meV can be observed for *W_D_* ≈ 500 nm, which is able to be distinguished by current transport experiments. Moreover, the non-uniformity, the different spin-flop structure as well as the curved shape of the DWs have little influence on the quantized transport of the layer filter; see the online supplementary material. Therefore, the dissipationless transport *G* = *e*^2^/*h* and the full layer polarization *P* = ±1, are robust against the irregularity of the DWs in $\rm {MnBi_2Te_4}$. Axion insulators have also been observed in topological insulators with magnetization deposited only on the top and bottom surfaces [85,86]. Our proposed layertronic devices can also be constructed by introducing domain walls in these systems.

### Layer valve

**Figure 3.**
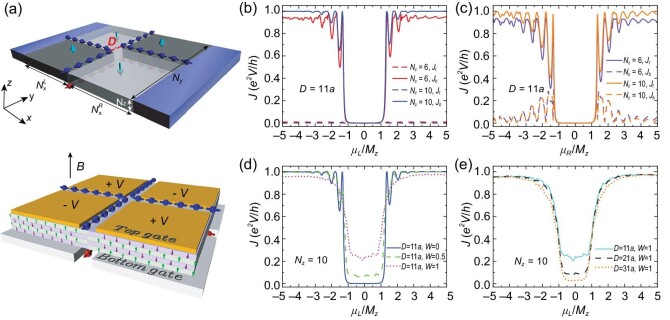
(a) Top panel: schematic of the layer valve. The DWs are separated by *D* in units of the lattice constant *a*. Bottom panel: the domain structure required by the layer valve can be realized in a gate-controlled way under a perpendicular magnetic field **B** [33]. (b and c) Layer-resolved currents *J_t_* and *J_b_* of the valve versus the TLF (BLF) chemical potential μ_*L*_ (μ_*R*_), with fixed μ_*R*_ = 0.02 (μ_*L*_ = 0.02) of the BLF (TLF), respectively. (d and e) The transmission current *J* = *J_t_* + *J_b_* in the presence of disorders versus μ_*L*_ (μ_*R*_ = 0.02) for different (d) *W* and (e) *D*. Model parameters are *N_y_* = 50, $N^{L}_{x}=N^{R}_{x}=50$ and the terminal chemical potential is 0.25.

The layer valve device serves as a switch to turn ‘on’ or ‘off’ the specific layer-polarized current. It is designed by connecting the TLF and BLF sequentially from left to right as sketched in Fig. 3(a). We denote by μ_*L*_ (μ_*R*_) the chemical potential of the TLF (BLF) in the top (bottom) part of the valve. Panels (b) and (c) of Fig. 3 show that the layer-resolved currents *J_t_* and *J_b_* [versus μ_*L*_(μ_*R*_) of the TLF (BLF)] both vanish in the gap of the surface states, indicating zero transmission current. The layer valve is in the ‘off’ status. This can be ascribed to the opposite chirality of the DW modes in the TLF and BLF [Fig. 3(a)]. The chirality reversal turns off the valve by blocking the transmission current on both the top and bottom layers. When we independently shift μ_*L*_(μ_*R*_) to the surface states, the layer valve is turned ‘on’ and generates current with bottom (top) layer polarization. As shown in Fig. 3(b), by shifting μ_*L*_, *J_b_* increases while *J_t_* remains at zero, indicating that the valve is turned on and works as a BLF. Similarly, when μ_*R*_ is tuned such that *E_F_* locates deeply inside the surface states, *J_b_* remains small while *J_t_* becomes significantly large [Fig. 3(c)], which implies that the valve is turned on as a TLF. Moreover, the quantized transport of *J_b_* and *J_t_* is better for thicker valves [see the curves in Fig. 3(b) and (c) with *N_z_* = 6 and *N_z_* = 10], which originate from the suppression of the backscattering between spatially separated DW modes.

The performance of the layer valve is also robust against disorders. As shown in Fig. 3(d), when μ_*L*_ is tuned such that *E_F_* lies inside the gap of the TLF and *W* < 0.5, *J* slightly deviates from zero, indicating that the layer valve is robust against weak disorders. For strong disorders, e.g. *W* = 1, *J* climbs up, leading to the reduction in the on off ratio. This stems from the scattering of DW modes in the TLF to that in the BLF under disorders. However, the on:off ratio can be significantly enhanced by separating the DW in the TLF and BLF further [increasing *D*; see Fig. 3(a)], which suppresses the scattering. In Fig. 3(e), *J* reduces as *D* increases; thus, the on off ratio rises accordingly. For spintronics/valleytronics, disorder induces large scattering between opposite spin/valley components, which greatly suppresses the on off ratio of the spin/valley valve [17,87]. In layertronics, besides the topologically protected chiral DW modes, the high performance of layer valve under disorder is also ensured by the spatially resolved layer degree of freedom. These transport properties are also verified by using realistic material parameters of $\rm {MnBi_2Te_4}$; see the online supplementary material.

Experimentally, the layer valve can be achieved in a gate-controlled way. As shown in Fig. 3(a), under a perpendicular magnetic field **B**, the applied gate voltages on the four split gates induce alternative electric field **E**, giving rise to the domain structure of the AFM order due to $\Delta \mathcal {L}=({\alpha \theta }/{4\pi ^2}) \mathbf {E}\cdot \mathbf {B}$ of the AI. Therefore, the layer valve devices with high on off ratio are experimentally feasible.

### Layer reverser

**Figure 4.**
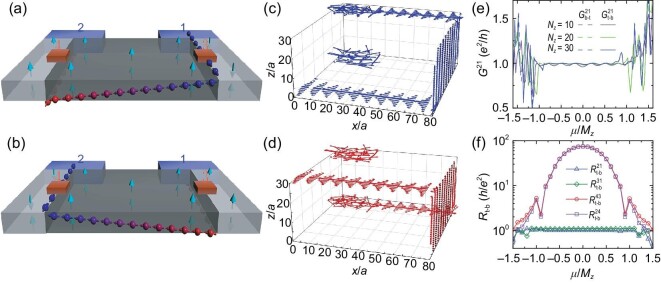
Schematics of top-bottom (a) and bottom-top (b) layer reversers. Colored arrows mark the layer reversing of the transmission modes. Terminals 3 and 4 are metallic contacts on the top surface. (c and d) Local current distributions of the two kinds of reversers (*N_z_* = 30), with μ = 0.02. (e) Differential conductance between terminals 1 and 2 of the top-bottom reverser $G^{21}_{\mathrm{t-b}}$ and the bottom-top reverser $G^{21}_{\mathrm{b-t}}$ versus μ with terminals 3 and 4 floated. (f) Local resistances of the top-bottom reverser versus μ. Model parameters are *N_y_* = 108, *N_x_* = 60, the terminal chemical potential is 0.2 and the sizes of contacts 3 and 4 are 8 × 8.

In applications, one expects that the binary bits stored inside the logical unit could be switched easily. However, switching internal degrees of freedom of electrons is challenging in experiments, especially for spintronics and valleytronics. An example is that the Datta–Das transistor [62–64], the reverser of the spin degrees of freedom of electrons, has not been completely realized and applied since its first proposal. A similar dilemma appears in valleytronics where an efficient valley reverser is hard to design. Interestingly, the feasibility of tuning the AFM AI $\rm {MnBi_2Te_4}$ into the FM-CI phase facilitates the layer reverser device, which inverts the layer degree of freedom dissipationlessly. As sketched in Fig. 4(a) and (b), the reverser consists of an FM-CI domain sandwiched by two AFM AIs with different AFM orders, which can be achieved experimentally by applying a vertical magnetic field on $\rm {MnBi_2Te_4}$ to reform the magnetization alignment [36,50].

The key ingredient that triggers layer reversing is the layer-crossing chiral edge mode, which starts from the top/bottom surface, goes through the CI edge mode on the side surface, then flows back into the bottom/top surface. The local current distributions [Fig. 4(c)] show that the top layer current is inverted to the bottom layer. We denote such a reverser as a top-bottom layer reverser. Similarly, the bottom-top reverser is realized by simply reversing the AFM order of the AIs [33] [Fig. 4(b) and (d)]. The dissipationless nature of the layer reverser is reflected by the quantization of the conductances between terminals 1 and 2 $G^{21}_{\mathrm{t-b}}$ and $G^{21}_{\mathrm{b-t}}$ for the two kinds of reversers. As shown in Fig. 4(e), in the gap of the surface states, $G^{21}_{\mathrm{t-b}}$ and $G^{21}_{\mathrm{b-t}}$ are nearly quantized (*e*^2^/*h*). As the thickness *N_z_* grows from 10 to 30, $G^{21}_{\mathrm{t-b}}$ and $G^{21}_{\mathrm{b-t}}$ are better quantized due to the suppression of the backscattering between two DW modes.

Experimentally, the reversal of layer polarization, as well as the information encoded by the layer degree of freedom, can be detected through the layer-resolved transport measurements. It can be achieved by attaching two additional point contacts (terminal 3 and 4) at the DWs on the top surface [Fig. 4(a–b)], and measuring the resistances between different pairs of terminals. As shown in Fig. 4(f), for the top-bottom reverser, colossal local resistances $R^{43}_{\mathrm{t-b}}$ and $R^{24}_{\mathrm{t-b}}$ appear around μ = 0 because of the absence of the transmission mode that connects terminal 4 with the others. In contrast, resistances $R^{21}_{\mathrm{t-b}}$ and $R^{31}_{\mathrm{t-b}}$ are quantized *h*/*e*^2^ because of the chiral mode that connects terminals 1 and 2 or 1 and 3.

## CONCLUSIONS

We introduced the layertronics and designed key devices utilizing the layer degree of freedom of the AFM AI $\rm {MnBi_2Te_4}$. The corresponding layer filter, layer valve devices were constructed based on the AFM-DW modes. The layer reverser device, which circumvents the difficulties in constructing reversers in spintronics and valleytronics, can be achieved through the FM-CI phase of $\rm {MnBi_2Te_4}$. The requirements for realizing layertronics are achievable under state-of-the-art experimental conditions. The robustness of DW modes under disorders realizes dissipationless manipulation of the layer degree of freedom, making layertronics a promising paradigm in constructing a new generation of electronic devices.

## Supplementary Material

nwad262_Supplemental_File which contain simulations with realistic material parameters and the proposal of realizing layertronic devices in a gate-controlled way, and include [69,88–97].
