# Supplementary material for: Dissipationless layertronics in axion insulator MnBi2Te4
Source: Natl Sci Rev. 2023 Oct 10;11(6):nwad262. doi: 10.1093/nsr/nwad262 (PMC11075771; doi:10.1093/nsr/nwad262)
Supplement: nwad262_Supplemental_File — which contain simulations with realistic material parameters and the proposal of realizing layertronic devices in a gate-controlled way, and include [69,88–97]. [file nwad262_supplemental_file.pdf]

Supplementary Materials for "Dissipationless Layertronics in Axion Insulator $MnBi_2Te_4$"

Shuai Li,[1,2,*] Ming Gong,[3,†] Shuguang Cheng,[4] Hua Jiang,[2,5,‡] and X. C. Xie[3,5,6]

[1] *School of Physical Science and Technology, Soochow University, Suzhou 215006, China*
[2] *Institute for Advanced Study, Soochow University, Suzhou 215006, China*
[3] *International Center for Quantum Materials, School of Physics, Peking University, Beijing 100871, China*
[4] *Department of Physics, Northwest University, Xi'an 710069, China*
[5] *Institute for Nanoelectronic Devices and Quantum Computing, Fudan University, Shanghai 200433, China*
[6] *Hefei National Laboratory, Hefei 230088, China*

CONTENTS

SI. Physical origin of the chiral domain wall modes in antiferromagnetic axion insulator $MnBi_2Te_4$ 1

SII. Simulation of the layertronic devices with the realistic material parameters of $MnBi_2Te_4$ 2

SIII. Influence of the domain wall structures 5
A. The finite-size effect of the DW 5
B. The influence of the non-uniformity and different spin-flop states inside the DW 6
C. The influence of the curved DW 7

SIV. Local current distributions in the layer valve 8

SV. Reversal of the layer polarization of the transmission currents in the layer valve 9

SVI. Layer valve with higher on-off ratio 10

SVII. Realizing layer filter and layer valve devices in a gate-controlled way 10

SVIII. Some details in the numerical calculations 11

References 11

SI. PHYSICAL ORIGIN OF THE CHIRAL DOMAIN WALL MODES IN ANTIFERROMAGNETIC AXION INSULATOR $MnBi_2Te_4$

In this section, we show the physical origin of the chiral domain wall (DW) modes in antiferromagnetic (AFM) topological axion insulator (AI) $MnBi_2Te_4$ in the view of Hall conductance. The two AFM ground states (I and II) of $MnBi_2Te_4$ are sketched in Fig. S1(a), where a magnetic DW is formed at the interface between them. We calculate the layer-dependent Hall conductance for AFM states I and II as functions of layer indices z [see Fig. S1(b)]. The Hall conductances on the inner layers ($z = 2, \cdots, 19$) are quantitatively small and oscillate around zero. While on the surface layers $z = 1$ and $z = 20$, the Hall conductances nearly approach $0.5(e^2/h)$ but with a sign reverse as labeled in Fig. S1(a), indicating that AFM $MnBi_2Te_4$ owns a half-quantized layer Hall effect where electrons on the top and bottom surface layers deflect in opposite directions [1]. We also evaluate the cumulative summation of the Hall conductance from each layer, i.e., $\sum_{n=1}^{n=z} \sigma_{xy}^{\mathrm{I/II}}(n)$, as given in Fig. S1(c). The cumulative conductances maintain almost half-quantized from $z = 1$ to $z = 19$ and vanish at $z = 20$, since the top and bottom surface layers cancel out each other. Therefore, the AFM AI $MnBi_2Te_4$ is characterized by a zero Hall conductance plateau [2]. We give the difference of the layer Hall conductances between the two AFM states, $\sigma_{xy}^{\mathrm{I}}(z) - \sigma_{xy}^{\mathrm{II}}(z)$, as a function of layer indices z in Fig. S1(d). On the surfaces ($z = 1, 20$), the conductance differences are quantized ($\pm e^2/h$), giving rise

* Shuai Li and Ming Gong are co-first authors
† Corresponding author: minggong@pku.edu.cn
‡ Corresponding author: jianghuaphy@suda.edu.cn

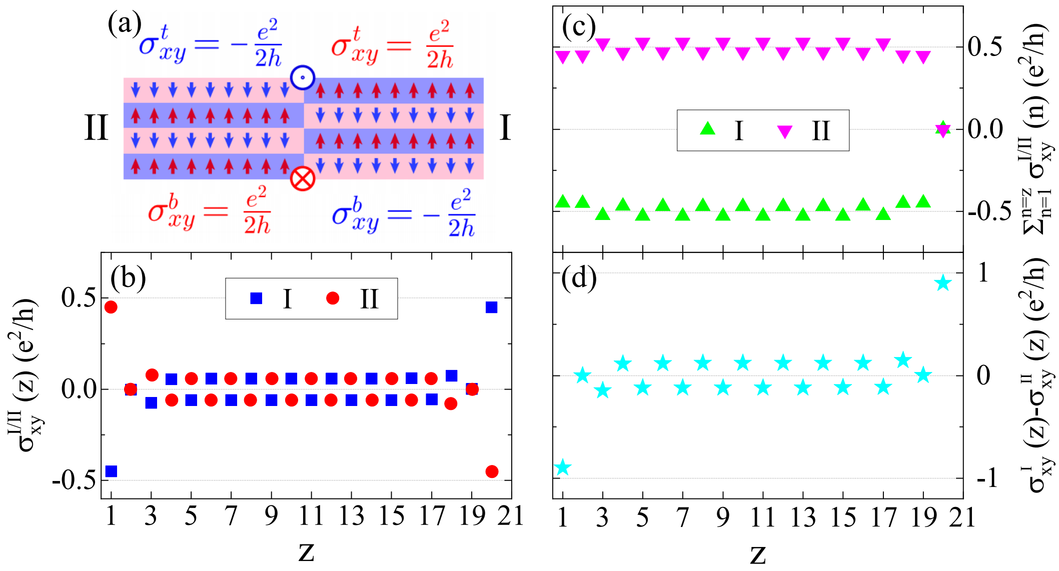

FIG. S1. (color online) (a) Sketch of the magnetic DW between the two AFM ground states I and II of $MnBi_2Te_4$. Blue and red arrows illustrate the magnetization in each single septuple layers. The vertices at the interface represent the propagation direction of the chiral DW modes. The half-quantized surface Hall conductances are labeled correspondingly. (b) Layer-dependent Hall conductances $\sigma^{I/II}_{xy}(z)$ of AFM states I and II, versus the layer index z. (c) The cumulative summation of the layer-dependent Hall conductances $\sigma^{I/II}_{xy}(z)$ from the 1st to the zth layer versus z. (d) Hall conductance difference between the two AFM states I and II as a function of z. Model parameters are: $M_z = 0.05$, $N_z = 20$ and Fermi energy of the AFM $MnBi_2Te_4$ $E_F = 0$.

to the generation of chiral modes at the DW between the two AFM domains. Inside the bulk ($z = 2, \cdots, 19$), the Hall conductance differences are nearly zero. It indicates that the chiral DW modes are spatially separated on the two surfaces. And the chirality of the DW modes is determined by the sign ($\pm$) of the Hall conductance differences on the surfaces and thus locked to the layer degree of freedom. Therefore, the DW modes have 1D features as illustrated in Fig. S1(a). Our numerical results give another view of the physical origin of the topological DW modes counter-propagating on different surfaces along the DW between the AFM $MnBi_2Te_4$ domains I and II.

SII. SIMULATION OF THE LAYERTRONIC DEVICES WITH THE REALISTIC MATERIAL PARAMETERS OF $MnBi_2Te_4$

The numerical calculations in the main text for the "layertronics" devices are based on a theoretical model with scaled parameters A_1, A_2, B_1, B_2, M_0 and perpendicular magnetization M_z (in unit of $\frac{\hbar V_F}{a}$ where V_F is the Fermi velocity of the Dirac surface states and $a = 1.35$ nm which measures the distance between two adjacent septuple layers of $MnBi_2Te_4$). To identify that our proposals are experimentally feasible in AFM AI $MnBi_2Te_4$, we give in this section the complementary calculations and discussions that utilize the realistic material parameters of $MnBi_2Te_4$.

An effective model for AFM $MnBi_2Te_4$ [3] is employed whose parameters are determined by fitting the energy spectrum of the effective Hamiltonian with that of the *ab* initio calculation. The effective material parameters are given as M_0=-0.1165 eV, A_1=2.7023 eV·Å, A_2=3.1964 eV·Å, B_1=-11.9048 eV·Å^2, B_2=-9.4048 eV·Å^2 [3–5]. Note that this effective model provides a reasonable perpendicular magnetization about M_z=0.04 eV along z direction in $MnBi_2Te_4$ septuple layers. Therefore, the surface band gaps of $MnBi_2Te_4$ induced by intrinsic magnetism can be large of about $2M_z$=0.08 eV, as consistent with the *ab* initio calculation [4]. The effective model is regularized on a cubic lattice with the lattice constant $a = 1.35$ nm standing for the distance between two adjacent septuple layers of $MnBi_2Te_4$.

The calculation results for the layer filter using realistic parameters are given in Figs. S2. The energy spectrum of the device exhibits a sizable surface gap of $\Delta \approx 2M_z$ =0.08 eV [see Fig. S2 (b)]. Inside the surface gap of the layer filter, two linear and layer-locked DW modes emerge. When the Fermi level E_F crosses over the surface states of the two leads and the DW states [see dashed line in Fig. S2(b)], a layer-polarized and quantized transmission current can be induced. Figure S2(c) plots the transmission current J and its layer polarization P versus the chemical potential μ of the filter. When E_F locates inside the surface gap or at the DW states ($-1 < \mu/M_z < 1$), a quantized current

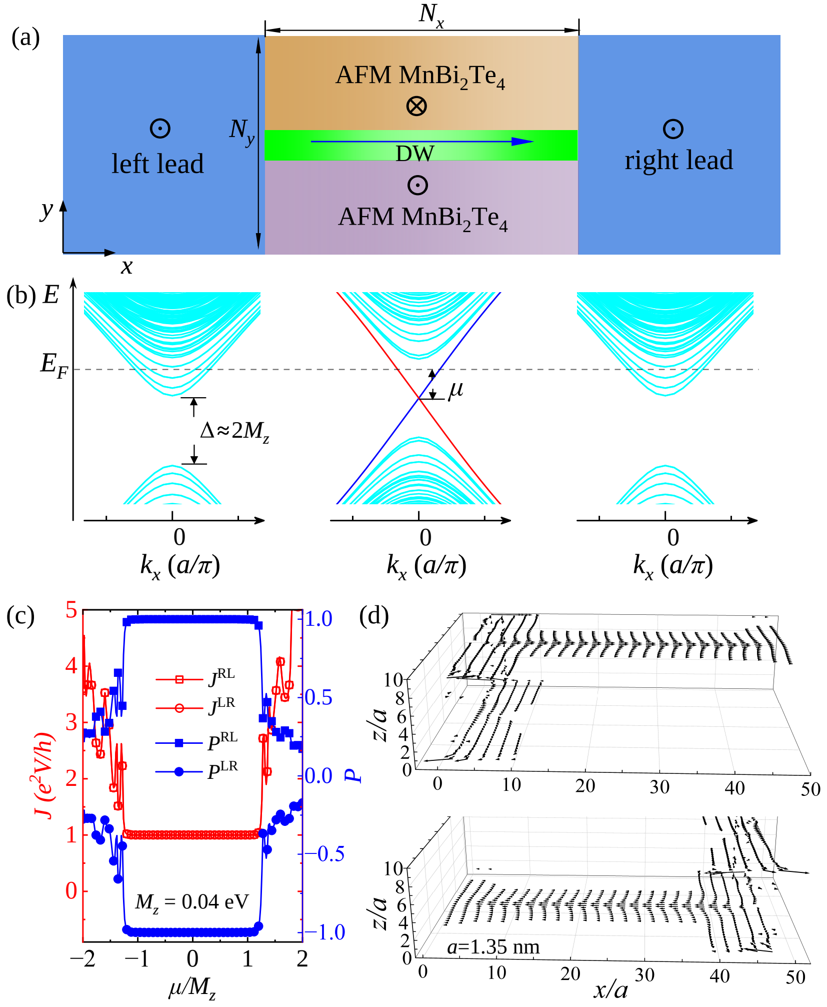

FIG. S2. (color online) (a) Top-view of the two-terminal layer filter device constructed with AFM $MnBi_2Te_4$. The vertices illustrate the directions (in and out) of the surface magnetization M_z. Horizontal blue arrow denotes the chiral DW mode on the top surface. (b) Corresponding energy spectrum of the leads and the filter. The blue/red linear dispersion highlights the DW mode on the top/bottom surface. μ is the chemical potential of the filter which measures the difference between Fermi energy E_F and the Dirac point. (c) Transmission current J^{RL} (J^{LR}) through the filter and its layer-polarization P^{RL} (P^{LR}) versus μ/M_z. The left/right (right/left) lead is the source/drain terminal. (d) Local current distributions of the top-layer (top panel) and bottom-layer (bottom panel) polarized currents through the layer filter, with $\mu/M_z = 0.25$. Other model parameters are: M_0=-0.1165 eV, A_1=2.7023 eV·Å, A_2=3.1964 eV·Å, B_1=-11.9048 eV·Å^2, B_2=-9.4048 eV·Å^2, M_z=0.04 eV, N_z=10, N_y=40, N_x=40, lead chemical potential $2M_z$=0.08 eV, and lattice constant a=1.35 nm (a measures the distance between two adjacent septuple layers of $MnBi_2Te_4$).

$J^{RL} = e^2V/h$ is flowing through with top-layer polarization $P^{RL} = +1$ [see the solid lines with empty and filled squares in Fig. S2(c)]. The filtered current can be directly visualized by the local current distribution, as shown in the top panel of Fig. S2(d). On the contrary, a bottom-layer polarized ($P^{LR} = -1$) current $J^{LR} = e^2V/h$ is transmitted through the filter by simply reversing the source and drain leads [see the solid lines with empty and filled circles in Fig. S2(c) and bottom panel of Fig. S2(d)]. The above calculations demonstrate that the layer filter constructed by $MnBi_2Te_4$ is capable of filtering electronic currents with specific layer-polarization. Therefore, we have theoretically proved the feasibility of fabricating the layer filter device with $MnBi_2Te_4$.

The layer valve device is designed with the AFM $MnBi_2Te_4$ by connecting the top-layer filter (TLF) and (BLF) from the left to the right, as sketched in Fig. S3(a). A sizable surface band gap Δ of about $2M_z$=0.08 eV is induced in the device by the intrinsic magnetism, as shown in the energy spectrum [see Fig. S3(b)]. Inside the gaps, a pair of linear dispersion appears both in the TLF and BLF. However, the chirality of the DW modes in the TLF and BLF on the same surface is opposite, as illustrated by the blue arrows in Fig. S3(a). The layer valve device can switch the layer-polarized currents on and off. As shown in Figs. S3(c) and (d), the top-layer current J_t, bottom-layer

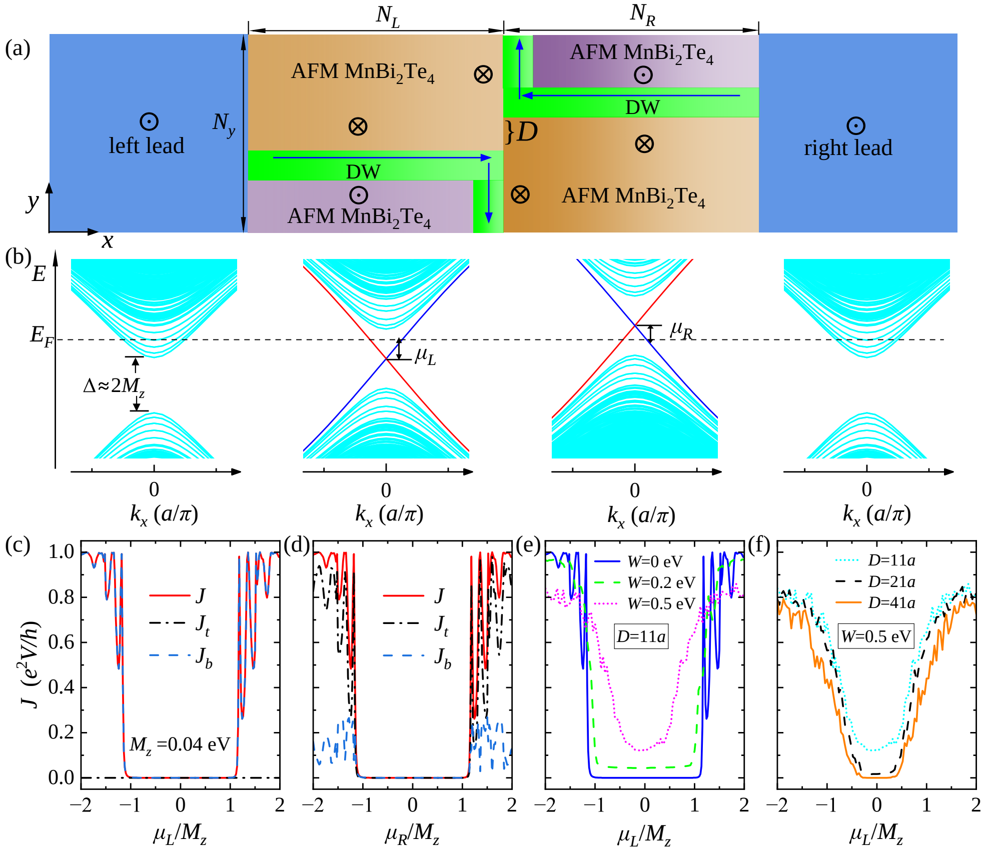

FIG. S3. (color online) (a) Top-view of the two-terminal layer valve device fabricated with AFM $MnBi_2Te_4$. The vertices represent the directions (in and out) of the surface magnetization M_z. Horizontal blue arrows denote the chiral DW modes on the top surfaces of the TLF and BLF. D is the separation of the two DW modes in y direction. (b) Corresponding energy spectrum of the leads and the valve. μ_L (μ_R) is chemical potential of the TLF (BLF) that measures the difference between Fermi energy E_F and the Dirac point of the DW modes in the TLF (BLF). The layer-resolved transmission currents J_t, J_b and $J = J_t + J_b$ through the valve versus (c) μ_L/M_z with $\mu_R/M_z = 0.25$ and versus (d) μ_R/M_z with $\mu_L/M_z = 0.25$. (e) Transmission current J through the valve versus μ_L/M_z with ($W = 0.2$ eV and 0.5 eV) and without (W=0 eV) disorders. $\mu_R/M_z = 0.25$. (f) Current J through the valve versus μ_L/M_z for $D = 11a$, $21a$ and $41a$, under disorders ($W = 0.5$ eV). $\mu_R/M_z = 0.25$. The left/right lead is the source/drain terminal. Other model parameters are: M_0=-0.1165 eV, A_1=2.7023 eV·Å, A_2=3.1964 eV·Å, B_1=-11.9048 eV·Å^2, B_2=-9.4048 eV·Å^2, M_z=0.04 eV, N_z=10, N_y=50, N_L=50, N_R=50, lead chemical potential $2M_z$=0.08 eV, and lattice constant a=1.35 nm.

current J_b and $J = J_t + J_b$ all vanish when Fermi energy E_F is located at the DW states of both the TLF and BLF ($-1 < \mu_L/M_z < 1$ and $-1 < \mu_R/M_z < 1$). The layer valve is tuned off. By independently shifting E_F out of the gap to the surface states of the TLF or BLF, a layer polarized current is generated with the layer valve switched on. Specifically, as shown in Fig. S3(c), by tuning E_F to the surface states of the TLF ($\mu_L/M_z < -1$ or $\mu_L/M_z > 1$), J_t keeps zero and J_b rises to e^2V/h, implying that a quantized and bottom-layer polarized current is switched on. Contrarily, the current with top-layer polarization can be induced by shifting E_F to the surface states of the BLF ($\mu_R/M_z < -1$ or $\mu_R/M_z > 1$), as shown in Fig. S3(d). From above, we demonstrate that the designed layer valve with AFM $MnBi_2Te_4$ can realize a switch on/off of the layer-polarized current with specific layer-polarization that is manipulated by independently tuning μ_L of the TLF or μ_R or the BLF.

The performance of layer valve is also robust against weak disorders. As shown by the green dashed line in Fig. S3(e), the transmission current J slightly deviates from zero when E_F sits both inside the surface gaps of the TLF and BLF (-1< μ_L/M_z <1 and -1< μ_R/M_z <1) under weak disorders W=0.2 eV, compared to that in the clean system (W=0 eV) shown by the blue solid line. However, the on-off ratio remains large since the scattering between the two DW modes of TLF and BLF is not much strengthened. Thus, the layer valve constructed with AFM $MnBi_2Te_4$ maintains functional and robust against weak disorders. In the presence of very strong disorders with W=0.5 eV (the surface gap approximates 0.08 eV), as shown by the pink dotted line in Fig. S3(e), J in -1< μ_L/M_z <1 climbs up to a moderate value, implying a leakage of the transmission current. Out of the surface gap, J cannot reach the

quantized value e^2V/h for that the tunneling electrons by the surface states of the TLF are scattered by disorders. Both aspects lower the on-off ratio of the layer valve. Whereas, we can enhance the performance of the layer valve by suppressing the scattering between the DW modes, which is realized by increasing their spatial separation D as shown in Fig. S3(a). When D rises from $11a$ to $21a$ and $41a$, the leaked current J lowers down to almost zero [see Fig. S3(f)]. Meanwhile, J in $\mu_L/M_z < -1$ or $\mu_L/M_z > -1$ is not much affected. The on-off ratio of the layer valve is then enhanced. We conclude that it is experimentally feasible to construct high performance layer valve device with the AFM $MnBi_2Te_4$.

SIII. INFLUENCE OF THE DOMAIN WALL STRUCTURES

In the main text, we have assumed that the magnetic DW between the two AFM AIs in the layer filter is atomically sharp. However, in experimentally grown AFM $MnBi_2Te_4$, the DW can be inherently wide [6] which may bring non-negligible finite-size effect. In addition, there is intrinsic spin structure within the DW, across which the magnetization smoothly varies to flip the spin order in the two domains of AFM $MnBi_2Te_4$ [6, 7]. Moreover, the shapes of the DWs can also be naturally curved as observed on the surfaces of $MnBi_2Te_4$ [6, 7]. We discuss, in this section, the effects of the finite-size, the spin-flop states, and shapes of the DWs on the transport properties of the layer filter device. Because of the topological origin of the DW modes, the layer-polarized and quantized transport is supposed to be robust and insensitive to the finite-size, the shapes and the spin structures of the DWs. Thus, it is achievable to realize the layer filter device in experiment with realistic materials, such as $MnBi_2Te_4$.

A. The finite-size effect of the DW

The schematic diagram of the layer filter is displayed in Fig. S4(a), where the boundaries of the AFM AIs form a smooth DW with finite width W_D. On the top/bottom surface, the perpendicular magnetization across such a DW is described by a smooth cosine function, i.e., $\pm M_z \cos[\frac{\pi}{W_D+2a}(y+\frac{W_D}{2}+a)]$, and the magnetization is homogeneous away from the DW, as shown in Figs. S4(a) and (b). In general, the finite W_D of the DW inevitably induces the trivial sub-band states on its surfaces whose gap E_g is related to W_D and can be approximated by $E_g = \frac{2\pi A_2}{W_D}$ if the DW has no spin structure inside ($M_z = 0$ across the DW) [8]. Here, A_2 is the Fermi velocity of the Dirac surface states in the DW. As W_D is increased large enough, the confinement induced sub-band gap E_g could decrease even lower than the intrinsic-magnetism raised surface gaps in AFM AIs (about $2M_z$), thus influencing the layertronic transport in the layer filter. That is to say, the dissipationless transport is possibly affected by the sub-band gap E_g. When taking the smooth spin structure of the DW into consideration, the sub-band gap E'_g should also exist.

In order to illuminate the finite-size effect of W_D with reduced computational resources, we first increase the magnetization M_z from 0.05 to 0.12 to create a larger magnetic surface gap. Then, we fix the width W_{AI} of the AFM AI, but only vary the DW width W_D, as show in Fig. S4(a). When W_D increases from 0 to $29a$ and $59a$, the sub-band gap of the DW states reduces [see Figs. S4(c)-(e)]. Especially as shown in Fig. S4(e), the sub-band gap ($E'_g \approx 1.125M_z$=0.135) is much smaller than the magnetic surface gap ($\approx 2M_z$=0.24), identifying the existence of the trivial 1D sub-band states in the DW. However, the topologically protected chiral DW modes persist in the sub-band gaps, independent of W_D [see green linear lines in Figs. S4(c)-(e)]. The effect of increasing W_D is barely broadening the DW states, as shown by the local density of states on the top and bottom surfaces in Figs. S4(f) and (g). The electronic transport currents by the DW modes preserve to be quantized ($J = e^2V/h$) and layer-polarized (P=+1)[see Fig. S4(h)], which clarifies the robustness of the layer filter against smooth domain walls. Nevertheless, the chemical potential μ-window where the layer filter works shrinks from the magnetic gap ($\approx 2M_z$) to the sub-band gap E'_g due to the finite-size effect of the DW, as shown in Fig. S4(h). We see above that the layertronic and quantized transport occurs in the sub-band gap E'_g of the DWs and thus determined by W_D. The larger W_D is, the narrower μ-window the layer filter works.

We further give an estimation of the critical W_D where the electronic transport by the DW modes can be distinguished in realistic material $MnBi_2Te_4$. Recall that the confinement-induced sub-band gap of the DW without spin structure is approximated by $E_g = \frac{2\pi A_2}{W_D}$. Taking the parameters of the effective model in Fig. S4(e), we have $E_g \approx 0.488M_z$=0.0586. From that $E_g < E'_g$, it is reasonably believed that the smooth domain wall spin structure renormalizes the realistic DW width by a factor α, i.e., $E'_g = \frac{2\pi A_2}{\alpha W_D}$. Then we obtain $\alpha = \frac{E_g}{E'_g} \approx 0.43$. The α is expected to be insensitive to the parameters if the spin structure insider the DW is smooth. Therefore it can be used as a constant to estimate the DW width with realistic material parameters. From the transport experiment of HgTe/CdTe[9, 10], it is proved that the topological transport can be detected as long as the bulk gap remains larger than about Δ=10 meV. Therefore for realistic $MnBi_2Te_4$, the DW width W_D is required to be smaller than $\frac{2\pi A_2}{\alpha\Delta} \approx$

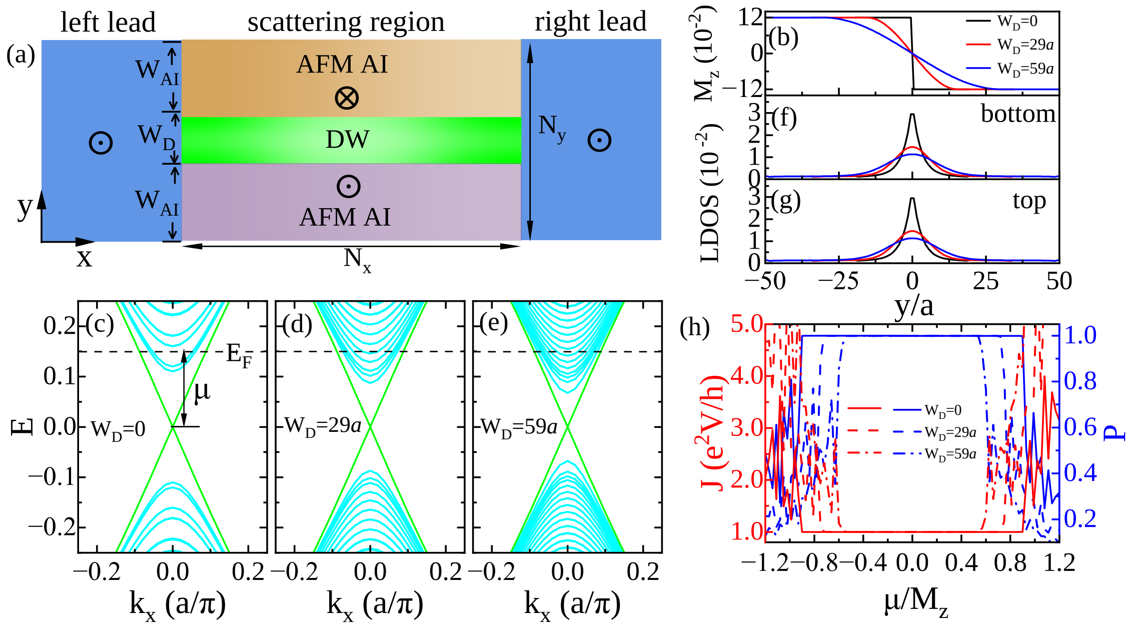

FIG. S4. (color online) (a) Top-view of the layer filter with smooth DW, whose width W_D is variable. The width W_{AI} of the AFM AI is fixed to 19a. a=1.35 nm measures the distance between two adjacent layers. (b) The top-surface perpendicular magnetization M_z of the layer filters with W_D=0, 29a and 59a. (c)-(e) Energy spectrum for the layer filters with W_D=0, 29a and 59a, respectively. (f)-(g) The bottom- and top-surface LDOS of the DW states for layer filters with W_D=0, 29a and 59a. The energy is E=0.01. (h) The transmission current J and its layer polarization P versus chemical potential μ of the layer filters with W_D=0, 29a and 59a. μ is defined as the difference between Fermi energy E_F and the Dirac point of the DW modes [see (c)]. Model parameters are: M_z=0.12, N_z=10, N_x=100, W_{AI}=19a and chemical potentials of the two leads 0.2.

468 nm ($A_2 \approx 3.2$ eV·Å is proportional to the Fermi velocity of the Dirac surface states for $MnBi_2Te_4$). We ensure that in such a condition, the layer filter keeps working and the layertronic transport can be detected. Note that the observed DW width in experiment is about a few hundred nanometers on the surfaces of $MnBi_2Te_4$ [6, 7], which quantitatively meets the requirement and makes $MnBi_2Te_4$ an ideal platform to construct layertronic devices.

The above estimation is based on the case that $MnBi_2Te_4$ is in clean condition. The disorder should exist in experimental samples. Meanwhile, the number of the trivial sub-bands around the DWs is small since they are spaced by $E'_g/2$ in energy regime. The 1D nature of the trivial states further makes them easier to be localized by disorders, expanding the μ-window where DW-mode transport occurs and the layer filter works. Therefore, quantized and layer-polarized transport can be observed in $MnBi_2Te_4$ with even wider DWs than the estimated 468 nm.

B. The influence of the non-uniformity and different spin-flop states inside the DW

The studies on the surface spin-flop transitions of the AFM $MnBi_2Te_4$ have demonstrated that the magnetic order within the DW is a canted AFM order [6, 7]. Besides the perpendicular component M_z, other two magnetization components (M_x and M_y) that are parallel to the terminated surface are possibly finite inside the DWs. To explore the effects of the DWs with realistic spin structures, some complementary calculations are performed and given in the Fig. S5. Two energetically favorable DW spin structures, the Néel wall and Bloch wall [11, 12], are considered. These two DW configurations can be described by a magnetization vector $\mathbf{M}(y) = (M_x, M_y, M_z) = M(\sin\theta\cos\phi, \sin\theta\sin\phi, \cos\theta)$, where M is a constant magnetization magnitude originating from magnetic doping [13, 14]. The azimuthal angle ϕ defines the type of DW. $\phi = 0$ and $\phi = \frac{\pi}{2}$ represent the Néel wall and Bloch wall respectively, as shown by the schematic diagrams in Figs. S5(a)-(b). And the azimuthal angle θ is a function of y with $\cos\theta(y) = -\tanh\frac{y}{W_D}$. The AFM magnetization is homogeneous away from the DW. We calculate the energy spectrum of the layer filters with the Néel and Bloch DWs [see Figs. S5(e)-(f)]. The DW modes emerge in the surface band gaps as expected, which originate from the topology difference across the DWs. The transmission currents through the filter carried by the DW states are quantized ($J = e^2V/h$) and layer-polarized ($P = +1$) [see red and blue solid lines in Figs. S5(i)-(j)], which evidently confirms that layer filters with the two DW structures are desirably

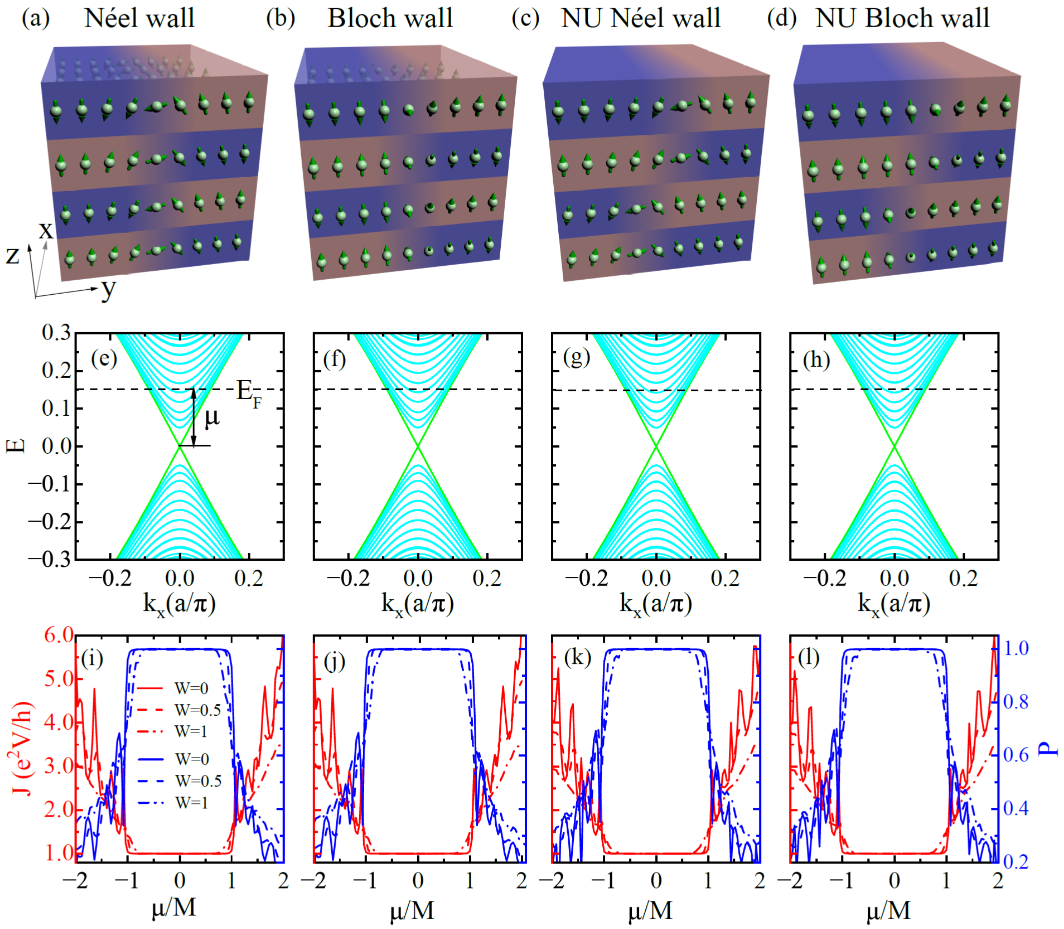

FIG. S5. (color online) (a)-(d) Schematic diagrams of the Néel, Bloch, nonuniform (NU) Néel and NU Bloch magnetic DWs (shadow regions). Green arrows with spheres illustrate the spin-flop on each layers. For Néel (Bloch) wall, the spin flops along y (x) direction. For NU Néel and NU Bloch walls, the magnetic DW on each layers is displaced randomly, as shown by the shadow regions. (e)-(h) Corresponding energy spectrum of the layer filters with the four kinds of DW structures. (i)-(l) The corresponding current J and layer polarization P versus chemical potentials μ/M, with (W=0.5, 1) or without (W=0) disorders. Model parameters are: M=0.05, N_z=10, N_y=60, N_x=80, W_D=19a and μ of the two leads is $4M = 0.2$. For the NU Néel and NU Bloch walls, the displacement of the magnetic DW on each layer is randomly chosen to be (5a 7a -6a 7a 2a -7a -4a 1a 8a 8a).

functional. When disorders are involved in, the transport properties ($J = e^2V/h$ and $P = +1$) persist owning to the topology and robustness of the DW states against disorders [see the red, blue dashed and dot-dashed lines in Figs. S5(i)-(j)].

Furthermore, we have also studied the non-uniformity of the DWs across the sample from the top to bottom layers. The magnetic DW on each layer is displaced randomly, as shown in Figs. S5(c)-(d). We plot the energy spectrum of the layer filters with nonuniform Néel and Bloch walls in Figs. S5(g)-(h). The non-uniformity brings little impacts and the DW modes in the surface gaps still exist. The characteristics of the electronic transport by the DW states with and without disorders are similar as that of the filters with uniform Néel and Bloch DWs [see Figs. S5(i)-(l)]. Thus, we conclude that the layer filter device can perform as well as designed, regardless of the spin structures, or the spin-flop states, inside the DWs between the AFM AIs.

C. The influence of the curved DW

The curved shape of the magnetic DWs is intrinsic in experimentally fabricated $MnBi_2Te_4$ [6]. We here simulate the curved domain wall by a cosine function $y = \frac{(N_y-1)a}{8}\cos(\frac{2\pi x}{L})$, as shown in the central part of Fig. S6(a). The topological DW modes are expected to emerge at the interface regardless of the shapes of the DWs. Their existence

is manifested by the energy spectrum where two linear dispersion crosses over the surface band gaps, as shown Fig. S6(b). We numerically plot the LDOS on the top and bottom surfaces of the chiral states inside the surface gap, as given in Figs. S6(d)-(e). The LDOS peaks nearby the curved interface, which further verifies that the chiral states are well-confined DW modes. The layer filter with curved DW shares similar electronic transport properties as the layer filter with straight DW by comparing their transmission current J and layer-polarization P, as shown in Fig, S6(c). Therefore, the layer filter with curved DWs can indeed filter quantized ($J = e^2V/h$) and fully layer-polarized ($P = +1$) currents. In conclusion, the layer filters maintain robust against curved DW structures.

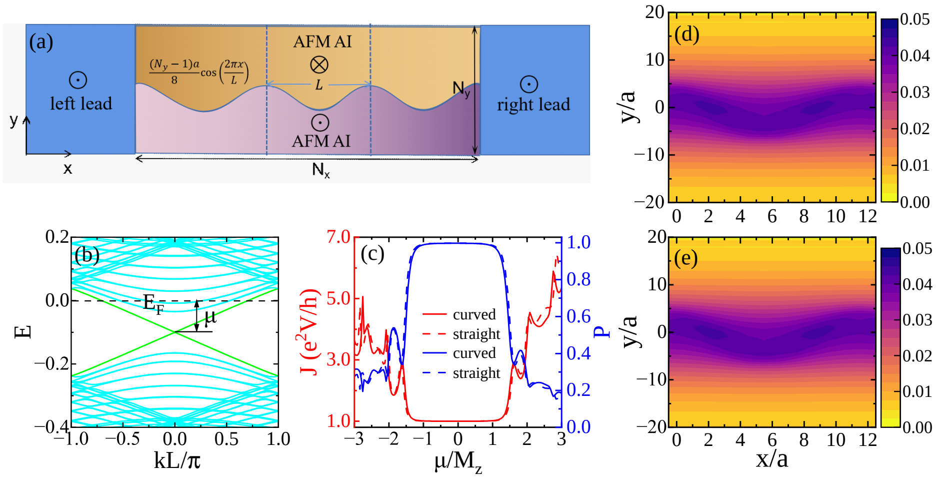

FIG. S6. (color online) (a) Top-view of the layer filter with curved DW described by $y = \frac{(N_y-1)a}{8}\cos(\frac{2\pi x}{L})$, where L is the period. The vertices represent the surface magnetization directions (in and out) of each part. (b) Folded energy spectrum of the layer filter. (c) The transmission current J and its layer polarization P of the layer filters with curved DW (solid lines) and straight DW (dashed lines), versus chemical potential μ/M_z. The left/right lead is the source/drain terminal. (d) Energy-resolved ($E = 0.01$) top and (e) bottom surface LDOS of the DW states inside the surface band gap. Model parameters are: $M_z = 0.05$, $N_z = 10$, $N_y = 40$, $N_x = 36$, $L = 12a$ and μ of the leads is $4M_z = 0.2$.

SIV. LOCAL CURRENT DISTRIBUTIONS IN THE LAYER VALVE

We have seen in the main text that the layer-polarized current can be switched on or off in the layer valve device, and its layer polarization is manipulated by independently controlling the Fermi level to the surface states of the TLF or of the BLF. We here display the local current distributions of the layer-polarized transmission currents when the valve is tuned on. For the first kind of "on" status, as shown in the bottom panel of Fig. S7(a), the current flows into the right terminal totally from the bottom surface with $P = -1$. The majority of electrons flowing to the interface ($x/a = 50$) between the TLF and BLF are reflected, because the number of surface state transverse modes in the TLF is significantly larger than that of the DW mode in the BLF [see top panel of Fig. S7(a)]. The local current distribution is determined by the interference of the N injected and $N - 1$ reflected current modes. The situation differs in the second case. As shown in the bottom panel of Fig. S7(b), the current flows to the right terminal entirely from the top surface ($P = +1$). The electrons carried by the upper DW mode of the TLF transmit to the top surface of the BLF without reflection, since only one injected mode in the TLF carries electrons to transport to the large N surface state transmitted modes in the BLF [see the top panel of Fig. S7(b)]. The local current distribution is determined by the perfect transmission mode.

The surface transport for the two "on" status of the layer valve is apparently different, as shown in Fig. S7. In spit of the mismatch between the number of transverse modes in the TLF and BLF discussed above, another aspect is the applied bias. Since a voltage drop is imposed between the leads to drive electrons to flow from left to right in both cases, there should be no symmetry connecting the surface transport in Figs. S7(a) and (b).

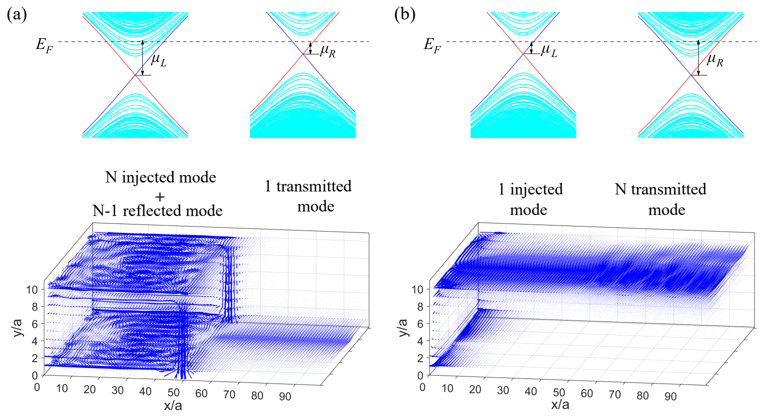

FIG. S7. (color online) (a) Top: Schematic energy spectrum for the TLF and BLF in the layer valve, with Fermi energy E_F marked by the dashed line. Chemical potential μ_L (μ_R) is the difference between E_F and the Dirac point of the DW modes in the TLF (BLF). Bottom: local current distribution of the bottom-layer polarized current in the layer valve, with $\mu_R/M_z = 0.4$ and $\mu_L/M_z = 4.1$. (b) Top: Schematic energy spectrum for the TLF and BLF. Bottom: local current distribution of the top-layer polarized current, with $\mu_L/M_z = 0.4$ and $\mu_R/M_z = 4.1$. Other model parameters are $M_z = 0.05$, $N_z = 10$, $N_y = N_x^L = N_x^R = 50$, $D = 11a$ and μ of the terminals is $5M_z = 0.25$.

SV. REVERSAL OF THE LAYER POLARIZATION OF THE TRANSMISSION CURRENTS IN THE LAYER VALVE

In this section, we show that by exchanging the positions of the TLF and BLF in the layer valve or by inverting the source and drain terminals in the main text, we are able to reverse and thus control the layer polarization of the transmission current when layer valve is tuned on. The newly structured layer valve is presented in Fig. S8(a). The chirality of all DW modes reverses on each part, compared to that in the main text [see Fig. 3(a)]. When either chemical potential μ_L of the left part (BLF) or μ_R of the right part (TLF) is located at the surface states to switch on the valve, the layer polarization of the transmission current is reversed as well, contrary to that in the main text [see Figs. 3(b)-(c)]. Specifically, as shown in Fig. S8(b), when μ_L is shifted from the DW states in the gap (-1< μ_L/M_z <1) to the surface states (μ_L/M_z <-1 or μ_L/M_z >1), the top layer transmission current J_t increases significantly from zero to about e^2V/h while bottom transmission current J_b keeps vanishing. A top-layer polarized current is generated [bottom-layer polarized current in the main text as shown in Fig. 3(b)]. On the other hand, when tuning μ_R, J_b arises from 0 to e^2V/h with vanishing J_t, as seen in Fig. S8(c). A bottom-layer polarized current is switched on [top-layer polarized current in the main text as shown in Fig. 3(c)]. The layer valve proposed here can generate layer polarized current whose layer polarization is opposite to that in the main text when shifting either μ_L of the left part or μ_R of the right part to the surface states. Note that the exchange of the TLF and BLF might be achieved using electromagnetic field to reverse the AMF order in each AI domain through the $\mathbf{E} \cdot \mathbf{B}$ term [1].

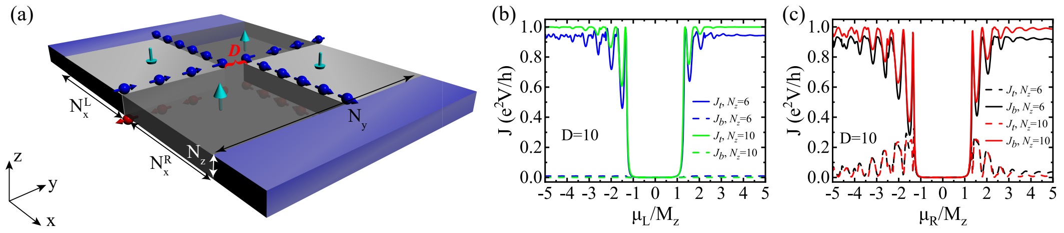

FIG. S8. (color online) (a) Sketch of the layer valve consisting of BLF and TLF that are connected in sequence from left to right. (b) Layer-resolved transmission currents J_t and J_b versus chemical potential μ_L/M_z of the left part (BLF) of the valve, with fixed chemical potential $\mu_R/M_z = 0.4$ of the right part (TLF). (c) J_t and J_b versus μ_R/M_z, with fixed $\mu_L/M_z = 0.4$. Model parameters are $M_z = 0.05$, $N_y = N_x^L = N_x^R = 50$, $D = 11a$ and μ of the leads is $5M_z = 0.25$.

SVI. LAYER VALVE WITH HIGHER ON-OFF RATIO

We propose that by inserting antiferromagnetic AI domain between the TLF and BLF of the layer valve in the main text [see Fig. 3(a)], the on-off ratio of the valve can be further enhanced in the presence of disorders, due to a larger separation and reduction of the scattering between the DW states in the TLF and BLF. This newly constructed layer valve is shown in Fig. S9(a). A central part of antiferromagnetic AI domain is inserted to separate the DW states further in x direction. As a consequence, the scattering between DW states in TLF and BLF assisted by disorders, is lowered and a higher on-off ratio is achieved compared to that in the main text. We plot together the transmission current J versus μ_L/M_z of this new valve and of the layer valve in the main text in the presence of disorders [see blue dashed and black dot-dashed lines in Fig. S9(b)]. When μ_L sits in the gap of the surface states (-1< μ_L/M_z <1), J of this new valve is smaller than that of the valve in the main text, indicating that this new layer valve possesses a higher on-off ratio in the presence of disorders.

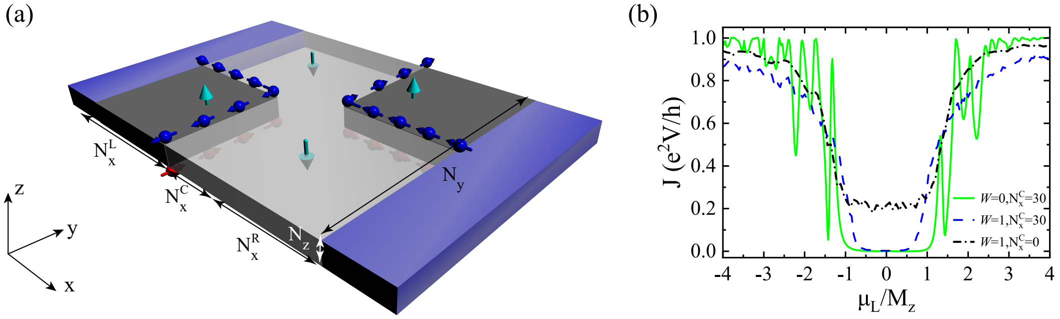

FIG. S9. (color online) (a) Schematic diagram of the layer valve consisting of TLF, AFM AI and BLF that are connected in sequence from left to right. (b) Transmission current J of this new valve (blue dashed line $N_x^C = 30$) and of the valve in the main text (black dot-dashed line $N_x^C = 0$) in the presence of disorders ($W = 1$), versus μ_L/M_z of the TLF and AFM AI, with fixed $\mu_R/M_z = 0.4$ of the BLF. Green solid line is the J of this new valve in absence of disorders, plotted for comparison. μ of the central AFM AI is set the same as that of the TLF. Model parameters are: $M_z = 0.05$, $N_z = 10$, $N_y = N_x^L = N_x^C = N_x^R = 30$ and μ of the leads is $5M_z = 0.25$.

SVII. REALIZING LAYER FILTER AND LAYER VALVE DEVICES IN A GATE-CONTROLLED WAY

In real experimental conditions, it may be difficult to realize the layertronic devices from naturally formed domain structures, especially the layer valve device. Fortunately, $MnBi_2Te_4$ is Van der Waals stacked material, of which the fabrication technique is based on mechanical exfoliation. Its fabrication technique is very similar to the bilayer graphene. Therefore, motivated by the technical route of realizing the valleytronic devices in bilayer graphene [15–19], we propose that the layer filter and layer valve devices can similarly be realized in a gate-controlled way. The key ingredient is that in the presence of a perpendicular magnetic field $\mathbf{B}$, the AFM order of $MnBi_2Te_4$ can be reversed by applying perpendicular electric field $\mathbf{E}$ due to the uique magnetoelectric coupling (i.e., the $\theta\mathbf{E}\cdot\mathbf{B}$) of the axion insulator. Such a process has already been realized experimentally [1]. Similar to what has been realized in bilayer graphene, the layer filter can be achieved by a pair of split gates on which opposite gate voltages are applied in the presence of a perpendicular magnetic field as shown in Fig. S10 (a). Due to the magnetoelectric coupling $\theta\mathbf{E}\cdot\mathbf{B}$, the domain of the perpendicular $\mathbf{E}$ gives rise to the domain of AFM order. Further, the layer valve device can be achieved by fabricating two pairs of split gates [see Fig. S10 (b)]. Moreover, the bulk gap of the valley valve devices is about 86 meV [17], while the surface gap of $MnBi_2Te_4$ is about 50-100 meV. They are of the similar order. Therefore, the topologically nontrivial transports should emerge at similar length scale of the devices.

There are some advantages in realizing layertronic deivces in $MnBi_2Te_4$ than realizing valleytronic devices in bilayer graphene. Firstly, the magnetization reversal can be achieved by applying an impulse voltage. Therefore, one convenience in $MnBi_2Te_4$ is that the gate voltage need not to be applied continuously. Specifically, the gate voltages applied on bilayer graphene should be fine-tuned such that the bulk gap of oppositely-gated domains are always aligned to ensure the global insulating bulk. But in $MnBi_2Te_4$, such a difficulty is naturally circumvented because we only need the impulse voltage to prepare the initial domain configuration. Secondly, during the repeatable switching process as shown in [1], the maximum (around +0.5 $k\Omega$) and the minimum values (around -0.9 $k\Omega$) of R_{yx} do not show any decay feature, which indicates that the reversal of the AFM order is of high-efficiency and causes little magnetization loss. Therefore, making high quality layertronic devices in $MnBi_2Te_4$ is achievable. Thirdly, the topological kink

states in valleytronics are not robust to disorders. This is because the chiral domain wall modes from K and K' valleys are not spatially separated and can be backscattered by short range disorders. Therefore, quantized transport is hard to observe. In contrast, the chiral domain wall modes in $MnBi_2Te_4$ are spatially separated (located on the top and bottom surfaces). Backscattering between these domain wall modes requires stronger disorder. Therefore, the quantized transport properties make the dissipationless layertronics easier to be characterized.

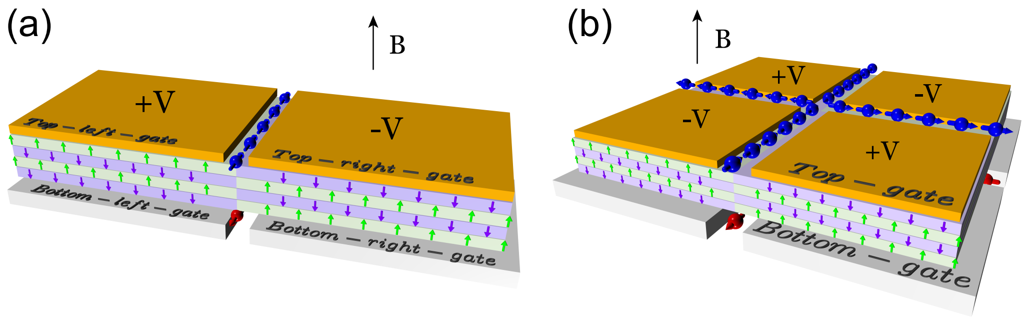

FIG. S10. (color online) Schematic of gate-controlled layer filter and layer valve devices in AFM $MnBi_2Te_4$. (a) The formation of a single domain wall to realize the layer filter can be achieved through a pair of split gates. Due to the magnetoelectric coupling of AIs, opposite gate voltages (V or $-V$) on the gates induce opposite perpendicular electric field $\mathbf{E}$, giving rise to a heterostructure of the two types of AFM orders under a perpendicular magnetic field $\mathbf{B}$. (b) The layer valve device can be similarly constructed by two pairs of split gates. Gate voltages are applied alternatively.

SVIII. SOME DETAILS IN THE NUMERICAL CALCULATIONS

This section is devoted to clarify some of the details in our numerical calculations in the main text. **1)** The NEGF method is applied for the calculation of local current distribution. The current flowing from site i to its neighboring site j can be calculated by [20]

$$J_{i\to j} = \frac{2e^2}{h}\mathrm{Im}\{\sum_{\alpha,\beta} H_{i\alpha,j\beta}\left[G^r\Gamma^L G^a\right]_{j\beta,i\alpha}\}(V_L - V_R), \tag{S1}$$

where $H_{i\alpha,j\beta}$ is the hopping of electrons from orbit α at i to orbit β at j, G^r (G^a) denotes the retarded (advanced) Green's functions of the central region, Γ^L represents the linewidth function of the left terminal, and $V = V_L - V_R$ gives the external bias. The layer-resolved transmission currents J_t, J_b, $J = J_t + J_b$ and layer polarization P are evaluated by summing $J_{i\to j}$ on a specific layer perpendicular to x direction that is close to the drain terminals, in order to explore the effects of layertronic devices on the unpolarized current injected from source terminals. **2)** Due to the restriction of the theoretical 3D topological insulator model, the Dirac fermions on the side surface are inevitably involved into the electronic transport. Experimentally, the contribution of the side surface states to the transport should not count, for that the size of the experimentally fabricated $MnBi_2Te_4$ is super large [e.g., see Fig. 1 in the main text. N_x and N_y are of the order μm, while N_z is of the order nm]. Thus in our calculations, we incorporate a y-direction magnetization term $M_2(y)s_2 \otimes \sigma_0$ in H_{mag} to gap out the side surface states. $M_2(y) = \pm M_y = \pm 0.3$ is the perpendicular magnetization on the side surfaces. **3)** We have shown the local current distributions for the layer filters and layer reversers in the main text. However, these configurations are plotted in a simplified way that the local currents whose magnitudes are smaller than a critical value, like 0.01 e^2V/h, are omitted for the sake of clarity. **4)** Disorder effect is also considered in the main text. The calculations for J and P are all averaged over 200 configurations of disorders. **5)** For the experimental scheme to verify the layer polarization reverse in the layer reverser device, we propose to attach two metallic contacts (terminals 3 and 4) at the DWs on the top surface. The density of states of the two metallic terminals are approximated by wide band approximation, and we choose the self-energy contributed by the two contacts as $\Sigma^r = -1.5i$ in our numerical calculations.

[1] A. Gao, Y.-F. Liu, C. Hu, J.-X. Qiu, C. Tzschaschel, B. Ghosh, S.-C. Ho, D. Bérubé, R. Chen, H. Sun, Z. Zhang, X.-Y. Zhang, Y.-X. Wang, N. Wang, Z. Huang, C. Felser, A. Agarwal, T. Ding, H.-J. Tien, A. Akey, J. Gardener, B. Singh,

K. Watanabe, T. Taniguchi, K. S. Burch, D. C. Bell, B. B. Zhou, W. Gao, H.-Z. Lu, A. Bansil, H. Lin, T.-R. Chang, L. Fu, Q. Ma, N. Ni, and S.-Y. Xu, Layer Hall effect in a 2D topological axion antiferromagnet, Nature **595**, 521 (2021).
[2] C. Liu, Y. Wang, H. Li, Y. Wu, Y. Li, J. Li, K. He, Y. Xu, J. Zhang, and Y. Wang, Robust axion insulator and Chern insulator phases in a two-dimensional antiferromagnetic topological insulator, Nat. Mater. **19**, 522 (2020).
[3] R. Chen, S. Li, H.-P. Sun, Q. Liu, Y. Zhao, H.-Z. Lu, and X. C. Xie, Using nonlocal surface transport to identify the axion insulator, Phys. Rev. B **103**, L241409 (2021).
[4] D. Zhang, M. Shi, T. Zhu, D. Xing, H. Zhang, and J. Wang, Topological axion states in the magnetic insulator $MnBi_2Te_4$ with the quantized magnetoelectric effect, Phys. Rev. Lett. **122**, 206401 (2019).
[5] R.-X. Zhang, F. Wu, and S. Das Sarma, Möbius insulator and higher-order topology in $MnBi_{2n}Te_{3n+1}$, Phys. Rev. Lett. **124**, 136407 (2020).
[6] P. M. Sass, W. Ge, J. Yan, D. Obeysekera, J. J. Yang, and W. Wu, Magnetic imaging of domain walls in the antiferromagnetic topological insulator $MnBi_2Te_4$, Nano Lett. **20**, 2609 (2020).
[7] P. M. Sass, J. Kim, D. Vanderbilt, J. Yan, and W. Wu, Robust A-type order and spin-flop transition on the surface of the antiferromagnetic topological insulator $MnBi_2Te_4$, Phys. Rev. Lett. **125**, 037201 (2020).
[8] Y. Yang, Z. Jia, Y. Wu, R.-C. Xiao, Z. H. Hang, H. Jiang, and X. Xie, Gapped topological kink states and topological corner states in honeycomb lattice, Sci. Bull. **65**, 531 (2020).
[9] B. A. Bernevig, T. L. Hughes, and S.-C. Zhang, Quantum spin hall effect and topological phase transition in HgTe quantum wells, Science **314**, 1757 (2006).
[10] M. König, S. Wiedmann, C. Brüne, A. Roth, H. Buhmann, L. W. Molenkamp, X.-L. Qi, and S.-C. Zhang, Quantum spin Hall insulator state in HgTe quantum wells, Science **318**, 766 (2007).
[11] G. Chen, J. Zhu, A. Quesada, J. Li, A. T. N'Diaye, Y. Huo, T. P. Ma, Y. Chen, H. Y. Kwon, C. Won, Z. Q. Qiu, A. K. Schmid, and Y. Z. Wu, Novel chiral magnetic domain wall structure in Fe/Ni/Cu(001) films, Phys. Rev. Lett. **110**, 177204 (2013).
[12] G. Chen, T. Ma, A. T. N'Diaye, H. Kwon, C. Won, Y. Wu, and A. K. Schmid, Tailoring the chirality of magnetic domain walls by interface engineering, Nat. Commun. **4**, 2671 (2013).
[13] K. Yasuda, M. Mogi, R. Yoshimi, A. Tsukazaki, K. S. Takahashi, M. Kawasaki, F. Kagawa, and Y. Tokura, Quantized chiral edge conduction on domain walls of a magnetic topological insulator, Science **358**, 1311 (2017).
[14] Y.-F. Zhou, Z. Hou, and Q.-F. Sun, Configuration-sensitive transport at the domain walls of a magnetic topological insulator, Phys. Rev. B **98**, 165433 (2018).
[15] L. Ju, Z. Shi, N. Nair, Y. Lv, C. Jin, J. Velasco, C. Ojeda-Aristizabal, H. A. Bechtel, M. C. Martin, A. Zettl, J. Analytis, and F. Wang, Topological valley transport at bilayer graphene domain walls, Nature **520**, 650 (2015).
[16] J. Li, K. Wang, K. J. McFaul, Z. Zern, Y. Ren, K. Watanabe, T. Taniguchi, Z. Qiao, and J. Zhu, Gate-controlled topological conducting channels in bilayer graphene, Nat. Nanotechnol. **11**, 1060 (2016).
[17] J. Li, R.-X. Zhang, Z. Yin, J. Zhang, K. Watanabe, T. Taniguchi, C. Liu, and J. Zhu, A valley valve and electron beam splitter, Science **362**, 1149 (2018).
[18] H. Chen, P. Zhou, J. Liu, J. Qiao, B. Oezyilmaz, and J. Martin, Gate controlled valley polarizer in bilayer graphene, Nat. Commun. **11**, 1202 (2020).
[19] I. Martin, Y. M. Blanter, and A. F. Morpurgo, Topological confinement in bilayer graphene, Phys. Rev. Lett. **100**, 036804 (2008).
[20] H. Jiang, L. Wang, Q.-f. Sun, and X. C. Xie, Numerical study of the topological anderson insulator in HgTe/CdTe quantum wells, Phys. Rev. B **80**, 165316 (2009).
